# A Unique Presentation of Bouveret's Syndrome: Two Large Gallstones Obstructing Both the Gastric Outlet and the Common Bile Duct Simultaneously

**DOI:** 10.1155/2021/8869803

**Published:** 2021-01-16

**Authors:** Yuqian Tian, Neha Sarvepalli, Mustafa Nazzal

**Affiliations:** ^1^Saint Louis University School of Medicine, St. Louis, MO, USA; ^2^Department of Surgery, SSM Health Saint Louis University Hospital, St. Louis, MO, USA

## Abstract

Bouveret's syndrome refers to a gastric outlet obstruction secondary to impaction of a gallstone in the pylorus or proximal duodenum. Thus, it can be considered a very proximal form of gallstone ileus and is infrequent. We describe such a unique case that a female patient presents with Bouveret's syndrome and concomitant common bile duct obstruction by a second gallstone. The decision over its surgical management is complicated, based on risk factors, clinical presentations, radiographic evidence, surgical risk assessment, and specific considerations tailored to individual case. Because of her stable clinical picture and low surgical risk, we proceeded with stone extractions, fistula take-down, and common bile duct exploration in a one-stage procedure. Her postoperative course was complicated by bile stained drainage through closed suction drain that resolved with conservative management.

## 1. Introduction

Gallstone ileus, a disease process defined as the passage of a gallstone through a bilioenteric fistula causing mechanical bowel obstruction, is one of the rare complications of symptomatic cholelithiasis (0.3% to 0.5% of patients with cholelithiasis) [1].

Various locations have been documented in the literature that are associated with gallstone ileus. The most common place of impaction is at the ileocecal valve due to its narrow lumen (60%), followed by jejunum (16%), stomach (15%), and colon (2-8%). The locations of obstruction are associated with the size of the stone and the size of the gastrointestinal (GI) lumen. Generally, a stone of greater than 2-2.5 cm in diameter is needed to cause obstruction in the GI tract. Few case reports have documented impaction of gallstone at sites of stricture or stenosis, associated with Crohn's, diverticulitis, and the neck of hernias [2, 3].

Bouveret's syndrome is one of the rare subtypes of gallstone ileus (1-3% of the cases). It describes gastric outlet obstruction caused by an impacting gallstone in the stomach or duodenal bulb.

We are presenting a unique case of Bouveret's syndrome with concomitant common bile duct obstruction with another large gallstone.

## 2. Case Presentation

A 60-year-old female with history of hypertension, type 2 diabetes, and history of deep vein thrombosis on chronic anticoagulation with warfarin, who was transferred from outside hospital (OSH), was presented for evaluation of cholecystoduodenal fistula.

**Figure 1:**
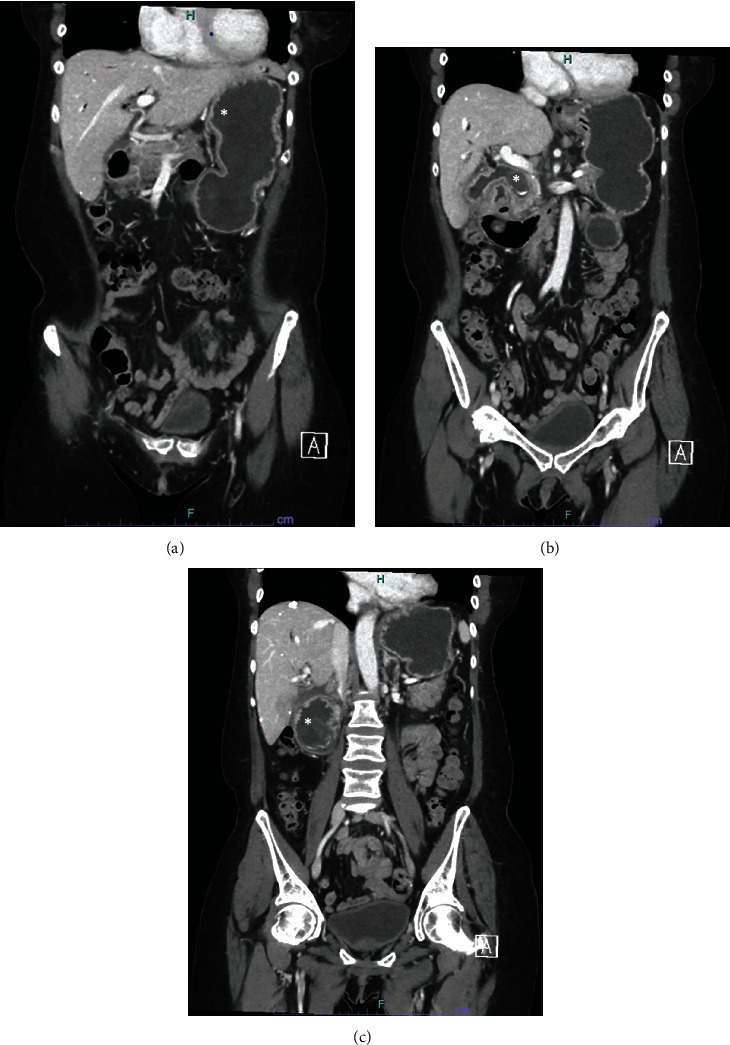
(a) CT A/P from OSH. The asterisk marks the dilated stomach from gastric outlet obstruction. (b) CT A/P from OSH. The asterisk marks the location of a 2.5 cm gallstone in the CBD. (c) CT A/P from OSH. The asterisk marks the location of a 3.5 cm gallstone in the 2^nd^ portion of duodenum.

**Figure 2:**
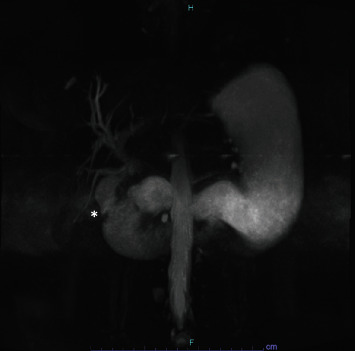
MRCP. Biliary anatomy and the fistula site (marked by the asterisk). The obstructing gallstone has migrated to the ligament of Treitz at this time.

Her initial presentation includes a 2-day history of right upper quadrant abdominal pain associated with nausea, dark bilious emesis, and the absence of flatus/bowel movements in the last 24 hours. Abdominal/pelvic computed tomography (CT) with contrast from OSH revealed cholecystoenteric fistula from the fundus of the gallbladder to the 2^nd^ portion of the duodenum with 3.5 cm gallstone within the duodenum, and with 2.5 cm gallstone within common bile duct (CBD) with extensive pneumobilia (Figure 1). Initial labs on presentation were unremarkable (total bilirubin -0.6 mg/dL, WBC −10.7 × 10^3^/*μ*L and Creatinine -0.8 mg/dL) except elevated alkaline phosphatase -336 units/L and INR -6.8. Upon her arrival, she underwent magnetic resonance cholangiopancreatography with double Eovist contrast and delays (MRCP) to help further delineate biliary anatomy, which revealed similar findings from the CT with the change of duodenal stone migrating to proximal jejunum and a better visualization of the fistula (Figure 2).

**Figure 3:**
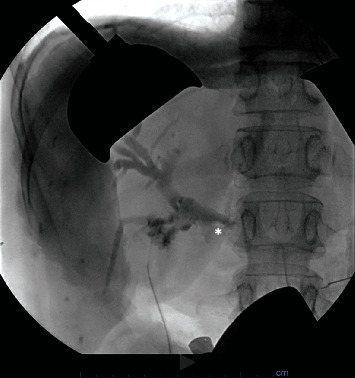
Intraop cholangiogram to delineate biliary anatomy. The asterisk marks the impacting CBD stone.

**Figure 4:**
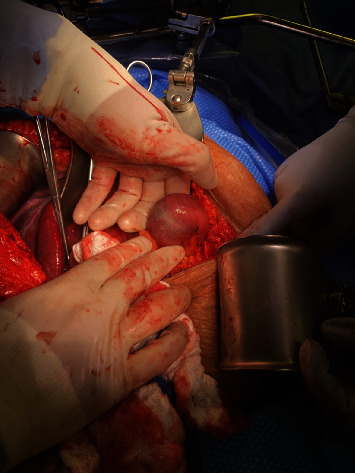
Intraop picture of the impacting gallstone in the proximal jejunum before extraction.

**Figure 5:**
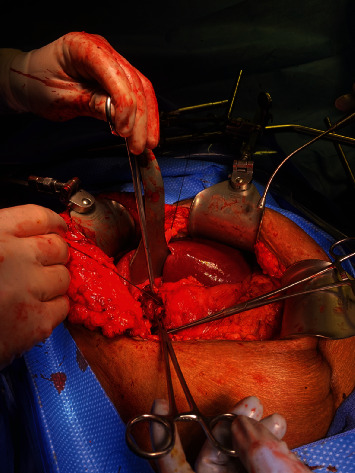
Intraop picture of the CBD after stone extraction. The CBD is marked by the probe.

**Figure 6:**
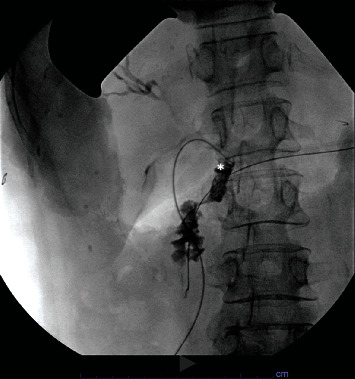
Intraop completion cholangiogram. It demonstrated good drainage with a patent CBD (marked by the asterisk).

**Figure 7:**
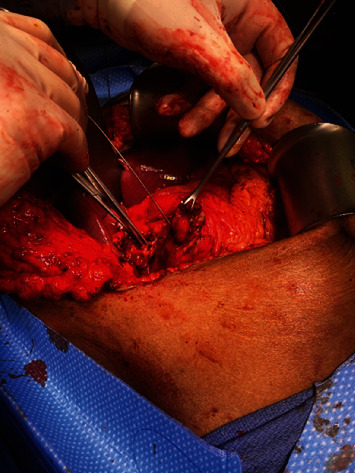
Intraop picture of the duodenal fistula before closure.

After appropriate resuscitation and gastric decompression, the patient underwent exploratory laparotomy, partial cholecystectomy, take-down of cholecystoenteric fistula, duodenal repair, jejunostomy with extraction of stone and primary closure, choledochotomy with common bile duct exploration and extraction of a large stone and closure using interrupted absorbable sutures, and intraoperative cholangiogram. Intraoperatively, the gallbladder was first opened, and an intraop cholangiogram was performed which delineated the CBD anatomy (Figure 3). Next, we identified one of the impacting gallstones at the proximal jejunum just distal to the ligament of Treitz (Figure 4). We performed enterotomy for stone extraction and closed the incision in the Heineke-Mikulicz fashion. Based on patient's presentation and risk factors, we decided to proceed with cholecystectomy and fistula take-down. A partial cholecystectomy was performed with anterior wall take-down due to severe inflammation and altered anatomy and concerns that otherwise will injure the CBD. When addressing the CBD impacting stone, we first attempted a transcystic approach to extract the stone but failed due to the size of the stone. Therefore, we performed a transverse choledochotomy, through which stone was extracted in pieces using the Randall stone forceps and was closed via interrupted absorbable sutures (Figure 5). A decision was made to not perform external drainage of the CBD with a T-tube, because the duct was large to 2.5 cm in diameter, and there was good drainage on completion cholangiogram (Figure 6). Lastly, fistula on the duodenum side was closed in 2 layers utilizing the Heineke-Mikulicz method (Figure 7). A JP drain was placed in the gallbladder bed near the duodenal fistula site before closure.

Pathology report confirmed the stones from the proximal jejunum and the CBD are 3.8 x 3.6 x 2.9 cm and 2.5 x 2.2 x 2.0 cm, respectively. Tissue from duodenal fistula showed mucosal erosion and acute inflammation with fibrosis on pathology report.

During her postoperative course, CT A/P with oral contrast was obtained on postop day (POD) 3 which revealed no obvious leak. Based on the unremarkable CT scan and benign clinical picture, we decided to start her on a clear liquid diet on POD 5. Immediately following diet change, JP drain acutely changed its content from serosanguinous fluid to dark bilious fluid. We suspected a leak from duodenal repair due to the extremely high amylase level detected in drain fluid. Because the patient at the time was clinically stable, no signs of peritonitis and sepsis, a repeated CT A/P with oral contrast on POD6 showed no extravasation of contrast from the duodenum, for which we proceeded with conservative medical management. Octreotide was started and the patient was maintained on nothing per OS along with starting total parenteral nutrition. On day 9 a nasojejunal tube distal to the duodenal suture line was inserted via esophagogastroduodenoscopy (EGD), during which we further evaluated for duodenal leak using contrast injection. EGD revealed inflammation in the second portion of duodenal wall opposite the major papilla without a good visualization of perforation or leak. The patient was started on enteral feeding with no issues.

HIDA scan was also performed and did not reveal a biliary leak. With consistent low JP drain output and negative scans, she was given a clear liquid diet trial again on POD13, and she tolerated diet well. The patient was discharged on POD15, tolerating regular diet, and having minimal JP drain output. JP drain was pulled out during a follow-up visit.

## 3. Discussion

The typical patient population of Bouveret's syndrome is elderly female, with a median age of 74 years and female to male ratio being 1.9. It is common for patients to have multiple comorbidities because of their advanced age at presentation, which explains the high morbidity and mortality rates associated with Bouveret's syndrome, 60% and 12-30%, respectively [4–6].

The diagnosis of Bouveret's syndrome is challenging. Its presentation usually consists of nonspecific signs suggesting upper GI obstruction: vomiting, nausea, and abdominal pain are the most common symptoms, followed by loss of appetite, weight loss, and anorexia [4]. The utility of abdominal X-ray (AXR) and ultrasonography (US) in Bouveret's syndrome is relatively limited. Therefore, the choice of noninvasive diagnostic test is computed tomography (CT). It has high sensitivity (90-93%) and specificity (100%) and can provide anatomical details that AXR and US cannot reveal. It allows for visualization of the fistulous connection, size of the gallstones, level of obstructions, and gallbladder anatomy, which can help to guide operative management [2, 7, 8].

Because of the unique patient population, some have advocated for endoscopic approach as first-line treatment to avoid high mortality and morbidity rate. However, it has many limitations, and stones that are larger than 2.5 cm are usually technically difficult to retrieve endoscopically. Because of the high failure rate (91%) of endoscopic approach, surgery is currently the most common intervention. Approximately 73% of patients undergo surgery for Bouveret's syndrome, among which 61% have failed endoscopy attempt prior [9–11]. The main controversy of surgical management lies between whether to address only the obstruction or both the obstruction and the fistula (one stage procedure). Treatment plan should be tailored to individual cases, including considerations of patient's age, comorbidities, and likelihood of recurrent biliary complications [12].

We describe a unique case of Bouveret's syndrome with combined intestinal and biliary obstructions by two separate large gallstones. ERCP was not a feasible option as the patient had symptoms of gastric outlet obstruction, and the impacted stones both in the duodenum and in the CBD were too large for endoscopic retrieval. A percutaneous transhepatic cholangiogram and drainage by interventional radiology to address biliary obstruction were also not an option because the patient had no signs of obstructive jaundice due to the presence of an alternative route of the bile through the cholecystoenteric fistula. Based on radiographic evidence and patient's presentation, the decision was to explore the patient surgically and to remove the gallstone causing the gastric outlet obstruction. We performed a magnetic cholangiopancreatography (MRCP) in addition to CT for preop planning to delineate the biliary anatomy due to the presence of CBD obstruction. Because of the size of the cholecystoduodenal fistula, we were concerned for recurrence of obstruction with a new stone; moreover, the CBD was completely obstructed and was impossible to be treated via ERCP due to the size of the stone; thus, there was also the risk of life-threatening cholangitis if the fistula closed spontaneously. Ultimately, we decided to perform a one-stage procedure to prevent serious complications. A two-staged procedure would have complicated the patient course extensively due to the aforementioned concerns of cholangitis and recurrent gastric/small bowel obstruction.

PubMed search showed one case report similar to our patient's presentation. It described a 42-year-old male presenting with Bouveret's syndrome and multiple common bile duct stones. After the failed endoscopy attempt, the patient underwent a partial duodenectomy, a gastrojejunostomy, a cholecystectomy, and a common bile duct exploration with extraction of multiple common bile ducts stones. A T-tube was placed into the bile duct in the end. The patient recovered appropriately without major complications [13]. It shared some similarities with our case, that one stage surgery was performed and that there were CBD stones present concomitantly in the context of Bouveret's syndrome.

Enterotomy with fistula closure in one stage is associated with higher risk of anastomotic leaks and intraabdominal abscess, comparing to enterotomy alone, in gallstone ileus cases [14]. In general, if patients present with contained leak and are relatively asymptomatic, medical or interventional treatment can usually successfully address the leak. Surgical management is only considered when patient is critically ill (with sepsis, peritonitis, and acidosis) with an uncontained leak or has failed medical and interventional management [15].

In our case, our patient had a contained bile leak during postop period. Because both CT and HIDA came back negative for leak and patient was clinically stable with no peritoneal or sepsis sign, we were confident that it was a contained small leak that we can manage with conservative measures. We kept the JP drain in place to continue draining the leak. We also started octreotide and attempted to place NJ tube distal to the suspected leak site for tube feeding. This management was proven to be adequate for our patient since she soon tolerated a diet, and her drain had minimum output and ultimately was removed.

## 4. Conclusion

We present a unique case of Bouveret's syndrome with concomitant CBD obstruction that complicated the surgical management. The patient underwent a one-stage procedure due to concerns of recurrence of obstruction and postoperative cholangitis. The patient had a successful outcome, with a minor bile leak that resolved with conservative management.
